# Characterising prescribing cascades in older community-dwelling adults attending general practice in The Netherlands: a retrospective cohort study

**DOI:** 10.1186/s12875-026-03273-x

**Published:** 2026-03-17

**Authors:** Ann Sinéad Doherty, Marieke Perry, Frank Moriarty, Fiona Boland, Barbara Clyne, Tom Fahey, Denis O’Mahony, Emma Wallace

**Affiliations:** 1 Department of General Practice, School of Medicine, University College Cork, Western Gateway Building, Cork, T12 XF62 Ireland; 2 Department of Primary and Community Care, Radboud University Medical Center, Nijmegen, The Netherlands; 3 School of Pharmacy and Biomolecular Sciences, RCSI University of Medicine and Health Sciences, Dublin, Ireland; 4 Data Science Centre, School of Population Health, RCSI University of Medicine and Health Sciences, Dublin, Ireland; 5 Department of Public Health & Epidemiology, School of Population Health, RCSI University of Medicine and Health Sciences, Dublin, Ireland; 6 Department of General Practice, RCSI University of Medicine and Health Sciences, Dublin, Ireland; 7 Department of Medicine, School of Medicine, University College Cork, Cork, Ireland; 8 Department of Geriatric and Stroke Medicine, Cork University Hospital, Cork, Ireland

**Keywords:** Potentially inappropriate prescribing, Prescribing cascades, Older adults, General practice, Electronic medical health records

## Abstract

**Background:**

Prescribing cascades, where one medication is used to treat/prevent the adverse effects of another, have been the subject of increased research focus. However, few studies have confirmed potential prescribing cascades identified via administrative or dispensed medicines records with general practice patient record data. This study examined the incidence of ThinkCascades, a list of nine prescribing cascades of clinical relevance in older adults developed via international expert consensus, using general practice data in The Netherlands.

**Methods:**

A retrospective cohort study examined prescribing records for adults aged ≥ 65 years captured within the FaMe-Net general practice database in The Netherlands for the period 2011–2021. The primary exposures were incident use of Drug A within each ThinkCascades dyad, with the primary outcomes defined as incident use of the corresponding Drug B within 365 days. Independent clinical review of identified potential prescribing cascades was conducted by a general practitioner and pharmacist using an approach consistent with methods applied in earlier prescribing cascade research.

**Results:**

The eligible cohort comprised 710 incident users of any ThinkCascades Drug A. Their mean age was 75.8 (SD = 6.1) years; 53.5% (*n* = 380) were female. Overall, 21 ThinkCascades dyads were identified in 18 participants, representing a one-year incidence proportion of 2.5% (*N* = 710). Only six of nine ThinkCascades were identified amongst study participants, most commonly the calcium channel blocker to diuretic prescribing cascade. Just over one-quarter of identified potential prescribing cascades (6/21; 28.6%) were supported as true prescribing cascades based on independent clinical record review. True cascade likelihood was indeterminable for three cases.

**Conclusions:**

Prescribing cascades, defined by ThinkCascades, were relatively uncommon over the eight-year study of older people attending Dutch general practice. Three of nine ThinkCascades were not identified. Only one in four identified prescribing cascades had evidence supporting a true prescribing cascade following independent clinical record review. Further research to characterise the prevalence of confirmed prescribing cascades is required. Recent research recommends expanding the number of prescribing cascade dyads which may impact identification rates. Tools to support prescribing cascade awareness, identification and deprescribing need to incorporate shared decision-making with patients and demonstrate clinical utility in supporting medication reviews in primary care.

**Supplementary Information:**

The online version contains supplementary material available at 10.1186/s12875-026-03273-x.

## Background

Addressing problematic polypharmacy in older adults remains a key international priority [1]. A biological vulnerability to adverse drug reactions (ADRs) set against a backdrop of increasing multimorbidity and polypharmacy places older adults at increased risk for medication-related harm [2–4]. It is estimated that up to 23% of hospital admissions in older adults are ADR-related, placing significant burden on healthcare systems, as well as the individual [5–7]. Consequently, tackling potentially inappropriate prescribing (PIP), where the potential benefits of medication are outweighed by their potential for harm, in older adults is warranted. One aspect of PIP that has garnered increased attention in recent years are prescribing cascades, with several scoping and systematic reviews published recently [8–11]. 

Prescribing cascades occur when the adverse effect of a medication are treated or prevented by prescribing another medication [12–14]. Prescribing cascades can occur intentionally and unintentionally and may also be further characterised as appropriate or inappropriate [15]. Prescribing cascades that occur unintentionally, via a misinterpretation of presenting symptoms as emergent illness, constitute an aspect of inappropriate prescribing as the patient remains exposed to the adverse effects of the first medication, with additional potential medication-related harm introduced by the subsequent medication [16]. For example, pedal oedema following initiation of a calcium channel blocker (CCB) may be misinterpreted as a sign of heart failure leading to diuretic prescribing.

Unintentional and inappropriate prescribing cascades may be more likely to occur in older adults due to the often-non-specific presenting nature of ADRs in this cohort [2]. For example, constipation, dizziness and fatigue are commonly reported ADRs which could have multiple other possible aetiologies. Older adults are also more likely to be living with multimorbidity, commonly defined as the presence of two or more long term conditions, and resulting polypharmacy, commonly used to refer to the use of five or more medications [3, 17, 18]. Increasing age, multimorbidity and polypharmacy are also known independent risk factors for ADR occurrence [19, 20]. 

Explicit lists of prescribing cascades of clinical importance in older adults have been developed by multidisciplinary expert consensus to aid prescribing cascade identification [21, 22]. Hypothesis-driven and hypothesis-free screening of routine administrative data sources has also identified novel signals of potential prescribing cascades [23–25]. However, much literature to date examining prescribing cascades in routine administrative data has been unable to proffer insight into whether identified signals constitute true prescribing cascades as opposed to prescribing for another clinical indication [23, 24, 26]. Indeed, few studies to date have conducted patient medical chart review to characterise ADR identification and subsequent prescribing. One study, that examined the calcium channel blocker to diuretic prescribing cascade, found that only 54.7% (*n* = 35) of patients identified via prescription sequence symmetry analysis (PSSA) (*N* = 64) were found to have a confirmed prescribing cascade on medical chart review, highlighting an important limitation of PSSA analyses and the value of reviewing the patient medical record to determine clinical indications for prescriptions [27]. 

Despite recognition that prescribing cascades may occur intentionally or unintentionally, no large-scale studies have reported on the prevalence of unintentional prescribing cascades. Furthermore, there is a paucity of studies that have examined prescribing cascades in general practice records, despite the wealth of data available in terms of diagnostic coding, clinical investigations, prescribing and referrals to secondary care. In many jurisdictions, general practitioners act as gatekeepers to specialist care in single-payer health systems [28]. Consequently, most prescribing and associated monitoring occurs in general practice, including the continued prescribing and monitoring of medications initiated by specialists in secondary care. Thus, general practice records may provide a more comprehensive data source within which to investigate prescribing cascades and to elucidate information regarding prescriber intent at point of prescribing.

This study aimed to examine the incidence of nine potential prescribing cascades, defined by the ThinkCascades list [21], amongst community-dwelling older adults attending general practice in The Netherlands. Furthermore, this study sought to explore evidence to support the plausibility of identified potential prescribing cascades via review of the patient’s electronic medical record.

## Method

The STrengthening the Reporting of Observational studies in Epidemiology (STROBE) statement guided reporting of this study (see Additional File 1) [29]. 

### Study design and setting

A retrospective observational cohort study using the Dutch Practice-Based Research Network (PBRN) Family Medicine Network (FaMe-NET) data was conducted [30]. FaMe-Net is a primary care health registration network affiliated with Radboud university medical center in Nijmegen, the Netherlands, and is the oldest uninterrupted PBRN in the world [30, 31]. In The Netherlands, individuals are registered to one family physician (general practitioner; GP) who provides medical care and acts as a gatekeeper for secondary care [31, 32]. For residents, GP care is typically free at the point of access, however residents are legally obliged to hold a standard healthcare insurance plan, which covers the associated cost [33]. FaMe-Net contains data from 35 GPs in six primary care practices and includes more than 42,000 patients [30]. For the year 2014, FaMe-Net contains data representing 3,997 patient-years for those aged 65 years and older [30]. FaMe-Net is representative of the general population in The Netherlands in terms of age and sex [32]. FaMe-Net practices are representative of GP practices in terms of average population size, ratio of male: female GPs and the proportion of practices that provide postgraduate GP training [34]. 

FaMe-Net collects data prospectively from participating GP practices. The database comprises anonymized patient data, with patients provided the opportunity to opt out of data extraction for research purposes [32]. In summary, data pertaining to demographics, GP-recorded medical diagnoses and consultations, clinical investigations, prescribed medications, and correspondence with other healthcare professionals is captured within FaMe-Net. Data validity is governed by regular meetings of the participating GPs to review, compare and discuss their classification and coding [31, 34]. System automation recognizes inconsistencies in registrations, with GPs alerted in cases of error [32, 34]. Systematic quality checks are conducted with feedback provided to GPs [32]. Prescription medications are coded in FaMe-Net using the World Health Organisation Anatomical Therapeutic Chemical (ATC) coding system [32]. Medical diagnostic coding is applied by GPs according to the International Classification of Primary Care 2nd Edition (ICPC-2) [32, 35]. 

### Study population

For the purposes of this study, nine separate analysis sets were created, representing each of the nine ThinkCascades [21]. A summary of the nine ThinkCascades, including medication ATC codes and cascade exclusions, is provided in Table 1. Participants aged 65 years and older and registered in FaMe-Net from 1st January 2011 to 31st December 2014 were included. The index date was defined as the date at which incident use of Drug A in each ThinkCascades dyad was prescribed. A washout period of 365 days was used to define incident use. The analysis sets were defined as follows.

**Table 1:** ThinkCascades dyads definitions

Exclusion (ICPC-2 code)	Drug A	ATC	Side Effect (ICPC-2 code)	Drug B	ATC
Diagnosis of heart failure (K77), previous history of peripheral oedema (K07)	Calcium channel blocker	C08	Peripheral oedema (K07)	Diuretic	C03
History of urinary incontinence (U02, U04)	Diuretic	C03	Urinary frequency, urgency, incontinence (U02, U04)	Overactive bladder medication	G04BD
History of Parkinson disease or other movement disorder (N87)	Antipsychotic	N05A	Extrapyramidal symptoms (N87)	Antiparkinsonian agent	N04
Existing cognitive impairment (P70)	Benzodiazepine	N05BA N05CD	Cognitive impairment (P70)	Cholinesterase inhibitor or memantine	N06DA N06DX01
Diagnosis of psychosis/schizophrenia (P72, P73), history of behaving irritably/angry (P04)	Benzodiazepine	N05BA N05CD	Paradoxical agitation or agitation secondary to withdrawal (P04)	Antipsychotic	N05A
History of sleep disorders (P06)	Selective serotonin reuptake inhibitors (SSRIs) Venlafaxine Vortioxetine	N06AB N06AX16 N06AX26	Insomnia (P06)	Benzodiazepines Benzodiazepine receptor antagonists Melatonin Doxepin Mirtazapine Trazodone Trimipramine Agomelatine Promethazine Clonazepam	N05BA N05CD N05CF N05CH01 N06AA12 N06AX11 N06AX05 N06AA06 N06AX22 R06AD02 N03AE01
History of hypertension or other indication for antihypertensive classification medication e.g., diabetes, myocardial infarction, nephrotic syndrome, heart failure, arrhythmias, anxiety, migraine prophylaxis, angina (K86, K87, T89, T90, K75, K80, K77, P74, N89, K74, K76)	NSAID	M01AE M01AB M01AC M01AG M01AH	Hypertension (K86, K87)	Antihypertensive	C02 C03 C07 C08 C09
Existing cognitive impairment (P70)	Urinary anticholinergics	G04BD02 G04BD04 G04BD06 G04BD07 G04BD08 G04BD09 G04BD10 G04BD11	Cognitive impairment (P70)	Cholinesterase inhibitor Memantine	N06DA N06DX01
History of postural hypotension (K88) or dizziness/vertigo (N17)	Alpha-1 receptor blocker	G04CA	Orthostatic hypotension, dizziness (K88, N17)	Betahistine Benzodiazepines Promethazine	N07CA01 N05BA N05CD R06AD02

- Participants aged ≥ 65 years
- Minimum of two years’ data in FaMe-Net (a minimum of one episode, contact or prescription in the year before and after the index date)
- Incident user of Drug A within a given ThinkCascades prescribing cascade
- Not received Drug B in the 365-day period prior to incident Drug A prescribing
- Not received a diagnosis of a condition for which Drug B could be prescribed prior to incident prescribing of Drug A

For those participants who met the above inclusion criteria, data pertaining to prescriptions from the 1^st^ January 2011 to 31^st^ December 2021 were available and examined, with the period of 1^st^ January 2011 to 31^st^ December 2011 used as a look back period to identify prevalent users. Thus, those participants who initiated Drug A in the years 2011 or 2021 were excluded to ensure sufficient look-back and follow-up data.

### Variables

**Fig. 1:**
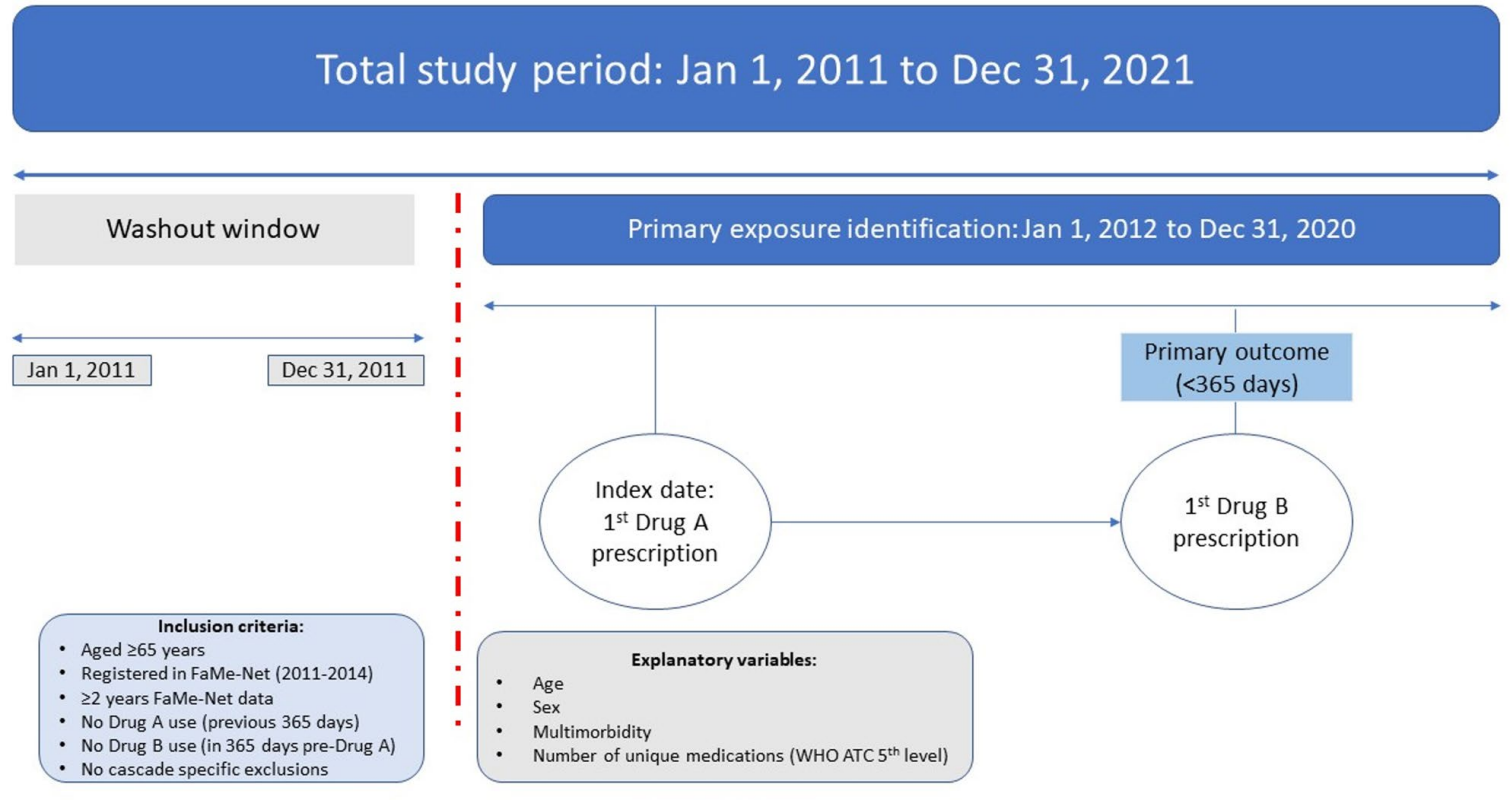
Overview of study design including ThinkCascades dyads washout window, inclusion criteria and outcome determination. Note: Drug A represents the medication hypothesised to cause the adverse drug reaction, with Drug B hypothesised as the corresponding treatment

The primary exposure was incident prescribing of Drug A in each ThinkCascades dyad, with the date of first prescribing defined as the index date (See Fig. 1). The outcome variable, a prescribing cascade, was defined as incident prescribing of Drug B, occurring after the index date and within 365 days of incident prescribing of Drug A, whilst still exposed to Drug A. Continuous exposure was calculated by combining successive prescriptions using the ‘drugprepr’ package. Participants were considered to be continually exposed to Drug A until the end date of their final Drug A prescription, with a maximum gap of 90 days allowed between successive prescriptions (i.e., between one prescription end and next prescription start) to account for medication non-adherence or delays in obtaining prescriptions.

Nine separate ThinkCascades prescribing cascades were examined representing nine different exposures and primary outcomes. However, for some ThinkCascades more than one medication class was explored as the exposure of interest and more than one medication class as the outcome of interest (see Table 1). Furthermore, the benzodiazepine to antipsychotic ThinkCascades dyad was specified in two ways to consider both possible ADRs as outlined in ThinkCascades, namely the occurrence of paradoxical agitation or agitation secondary to withdrawal. To examine agitation secondary to withdrawal, a discontinuation cascade, case identification was conducted differently. Further detail on the specification of this discontinuation cascade is provided in Additional File 2. Furthermore, a composite variable, any ThinkCascades-defined potential prescribing cascade, was defined as the occurrence of any of the nine ThinkCascades dyads (excluding the benzodiazepine discontinuation cascade).

Additional explanatory variables of interest included age, sex, multimorbidity and number of prescribed medications, assessed at the index date. Age was calculated as age in the index year (year of incident Drug A) based on birth year. Multimorbidity was defined as the presence of two or more long term conditions from a list of 36 ICPC-2 coded conditions specified in Additional File 3 up until the index date. The number of prescribed medications was defined as the total number of unique medications, defined at the ATC 5th level, prescribed in the year prior to the index date.

### Data analysis

Descriptive statistics were generated to describe the sample at the index date. In per protocol analyses, the incidence of prescribing cascades was defined as the number of participants who met the outcome definition (numerator) divided by the number of incident users of Drug A (denominator) during the period 1st January 2012 to 31st December 2020. A sensitivity analysis was conducted using an intention to treat approach, whereby the outcome, a prescribing cascade, was defined as initiation of Drug B within 365 days of Drug A, without specifying continued exposure to Drug A. All available prescriptions recorded within the database were analysed, irrespective if they were prescribed directly by the patient’s GP or were prescriptions issued by another prescriber, subsequently dispensed by a pharmacy, and transferred electronically to the GP database via the national medication record system. Analyses were conducted using R version 4.3.2 via the R Studio 2023.12.0 (Build 369) interface and using the “drugprepr” package (version 0.0.04) to specify medication exposure periods.

The anonymized electronic health record data for all participants who were identified as experiencing a potential prescribing cascade were further examined. This review examined the episodes of care data and aimed to evaluate whether the prescribing of Drug B was consistent with treatment of an ADR induced by Drug A or if it was prescribed for another clinical indication. Within an episode of care record all information related to the same health problem, including hospital reports, is linked and ordered from beginning to end. This way all information related to this presentation can be viewed chronologically to illustrate the developing clinical scenario [32]. The records of incident prescribing of both Drug A and Drug B were examined to identify the related episode of care number and cross-referenced to identify the associated clinical diagnostic codes applied (ICPC-2 codes). The application of a diagnosis code within the same episode of care in which the medication was initiated or applied to the record on the same date that medication was initiated was deemed to be the reason for prescribing. Consequently, when prescribing of both Drug A and Drug B occurred within the same episode of care and/or related to the same diagnostic code, prescribing for Drug B was deemed not to be for the treatment of an ADR induced by Drug A.

Two independent raters (GP and pharmacist) reviewed each case (EW, ASD) and classified each on a five-point adjective scale regarding likelihood of a true prescribing cascade: definitely no; unlikely; probably; definite yes; unable to determine, in line with previously published literature [27]. Reviewer ratings were then further categorized as Yes (Prescribing Cascade: probably or definitely yes) or No (Prescribing Cascade: definitely no, unlikely, unable to determine). No cases required referral to a third independent reviewer for adjudication. The percentage of cases classified as true prescribing cascades, representing the positive predictive value, was expressed as the number of those rated ‘Yes’ as a proportion of all identified potential prescribing cascade cases. The independent raters also assessed intentionality for true prescribing cascades, categorized as ‘intentional’ or ‘unintentional’.

### Ethical approval

Studies using FaMe-Net data are exempt from ethical review by the Dutch Central Committee on research involving human subjects (CMMO) [34, 36–38]. Under the FaMe-Net system, patients are informed of the use of their health-related information in FaMe-Net and may opt-out of participation [30, 32, 36, 37]. In Ireland, ethical approval for this study was obtained from the Clinical Research Ethics Committee (CREC) of University College Cork (ECM 4 (d) 24/10/2023; ECM 3 (oo) 04/02/2025). Data routinely collected by FaMe-Net was submitted to the Radboudumc local ethics committee for review and they declared that formal judgement was not required according to Dutch law (number 2020–6871).

## Results

### Participant characteristics

Descriptive statistics at baseline (index date) for the total eligible sample are presented in Table 2. A total of 710 participants were incident users of Drug A in any ThinkCascades dyad. Just over half (53.5%, *n* = 380) of the sample were female and more than two-thirds had multimorbidity (69.6%, *n* = 494). More than half of those who experienced any ThinkCascades were female (55.6%, *n* = 10) and more than three-quarters had multimorbidity (77.8%, *n* = 14).

**Table 2:** Descriptive statistics-study population overall and by any and no ThinkCascades occurrence

Characteristics	Overall *N* = 710 *n* (%)	Any ThinkCascades *N* = 18 *n* (%)	No ThinkCascades *N* = 692 *n* (%)
Age (years), *M (SD)*	75.8 (6.1)	75.2 (5.8)	75.8 (6.1)
Sex			
Male	330 (46.5)	8 (44.4)	322 (46.5)
Female	380 (53.5)	10 (55.6)	370 (53.5)
Multimorbidity			
0 chronic conditions	84 (11.8)	2 (11.1)	82 (11.8)
1 chronic condition	132 (18.6)	2 (11.1)	130 (18.8)
≥ 2 chronic conditions	494 (69.6)	14 (77.8)	480 (69.4)
Number of unique prescriptions (ATC 5th level)	6.6 (5.2)	7.1 (5.1)	6.6 (5.2)

### Primary outcome

A total of 21 potential prescribing cascades affecting 18 patients were identified for six of the nine ThinkCascades dyads during the nine-year observation period, representing an overall incidence of 2.5% (*N* = 710). One additional case was identified for the benzodiazepine discontinuation leading to antipsychotic initiation cascade (*N* = 262, 0.4%). One participant was prescribed two potential prescribing cascades, and another participant was prescribed three potential prescribing cascades.

The CCB to diuretic prescribing cascade was most common, with 13 cases identified amongst 141 CCB users, representing an incidence proportion of 9.2% (see Table 3). Time from CCB initiation to diuretic initiation ranged from seven to 364 days. Three patients prescribed benzodiazepines were subsequently prescribed antipsychotics within 365 days, an incidence of 1.1% (*n* = 263). Time to this cascade ranged from eight to 139 days. Two diuretic users (of *n* = 215) commenced an overactive bladder medication between 29 and 206 days; one patient (of *n* = 269) prescribed benzodiazepines commenced an anti-dementia medication after 139 days; one patient (of *n* = 19) prescribed a selective serotonin reuptake inhibitor (SSRI)/selective noradrenaline reuptake inhibitor (SNRI) commenced a sleep agent after 47 days; and one patient (of *n* = 84) prescribed an alpha-1-receptor blocker commenced a vestibular sedative within 94 days. No cases were identified for the prescribing cascades of antipsychotic to antiparkinsonian drug, NSAID to antihypertensive drug or urinary anticholinergic to anti-dementia drug.

**Table 3:** Incidence of ThinkCascades from 1^st^ January 2012 to 31^st^ December 2021: per protocol and intention to treat analyses

ThinkCascades dyad		Per protocol analyses			Intention to treat analyses		
	Incident users Drug A (*n*)	Prescribing cascade (*n*)	Incidence (%)	Days to cascade	Prescribing cascade (*n*)	Incidence (%)	Days to cascade
Calcium channel blocker (CCB) to diuretic	141	13	9.2	7-364	20	14.2	7-364
Diuretic to overactive bladder medication	215	2	0.9	29–206	4	1.9	29–206
Antipsychotic to antiparkinsonian medication	54	0	-	-	0	-	-
Benzodiazepine to anti-dementia medication	269	1	0.4	139	2	0.7	139–248
Benzodiazepine to antipsychotic	263	3	1.1	8-139	4	1.5	8-346
SSRI/SNRI to sleep medication	19	1	5.3	47	1	5.3	47
NSAID to antihypertensive	123	0	-	-	7	5.7	85–362
Urinary anticholinergic to anti-dementia medication	80	0	-	-	1	1.2	258
Alpha-1-receptor blocker to vestibular sedative	84	1	1.2	94	4	4.8	94–186

### Electronic health record validation

Corresponding electronic health record review was conducted for all participants who were identified as having a potential prescribing cascade, except for the benzodiazepine withdrawal induced cascade (*n* = 18 participants). Across 21 potential cascades (*n* = 18 participants), assessments by two independent reviewers showed 100% agreement. For three potential cascades (14.3%), the adjudicators were unable to make a determination on cascade likelihood due to missing data.

Among the 21 potential prescribing cascade cases reviewed, six (28.6%) were determined to be a true prescribing cascade (probable or definite yes). Of these six true cascades, the independent raters deemed four to be unintentional prescribing cascades, with insufficient information available for the remaining two cases for prescriber intent to be inferred. For these six cases, where intentionality was rated, the two independent reviewers’ assessments were in 100% agreement. The positive predictive value for each case ranged from 0% for the benzodiazepine to anti-dementia medication and alpha-1-receptor blocker to vestibular sedative cascades, to 100% for the diuretic to overactive bladder medication cascade (see Table 4). Of the 13 potential CCB to diuretic prescribing cascades identified, the diuretic was initiated for hypertension in six cases. For those participants who were identified as experiencing multiple potential prescribing cascades (*n* = 2), only one participant was adjudicated as a confirmed prescribing cascade (diuretic to overactive bladder medication) which was deemed to be an unintentional prescribing cascade.

**Table 4:** Percentage of potential ThinkCascades dyads (21 cascades, across 18 participants) confirmed via corresponding patient medical record review validation

ThinkCascades dyad	Positive predictive value % (*n*)^a^
CCB to diuretic^b^	23.1 (3)
Diuretic to overactive bladder	100 (2)
Antipsychotic to antiparkinsonian	-
Benzodiazepine to anti-dementia	0 (0)
Benzodiazepine to antipsychotic^c^	33.3 (1)
SSRI/SNRI to sleep agent^d^	0 (0)
NSAID to antihypertensive	-
Urinary anticholinergic to anti-dementia	-
Alpha-1-receptor blocker to vestibular sedative	0 (0)

^a^ Expressed as a proportion of the number of cascade instances

^b^ Unable to determine likelihood for one potential case

^c^ Unable to determine likelihood for one potential case

^d^ Unable to determine likelihood for one potential case

## Discussion

### Principal findings

The incidence of potential prescribing cascades, as defined by ThinkCascades, was low over the eight-year study period, with an overall one-year incidence proportion of 2.5% across any ThinkCascades dyad. Six of nine ThinkCascades dyads were identified over the study period. The most commonly identified prescribing cascade was the CCB to diuretic cascade, with almost one in ten incident CCB users found to also initiate a diuretic within the subsequent year (9.2%). Other ThinkCascades dyads identified included: (i) SSRI/SNRI to sleep medication (5.3%); (ii) alpha-1-receptor blocker to vestibular sedative (1.2%); (iii) benzodiazepine to antipsychotic (1.1%); (iv) diuretic to overactive bladder medication (0.9%); and (v) benzodiazepine to anti-dementia medication (0.4%). Independent review of the 21 potential incidences of prescribing cascades in 18 participants after applying the nine ThinkCascades dyads and the corresponding patient electronic health records determined that just over one-quarter (*n* = 6; 28.6%) were true prescribing cascades, with four of these deemed to be unintentional prescribing cascades. True prescribing cascade likelihood could not be determined for three cascades (14.3%) due to missing data. Similarly, prescriber intent could not be determined for two prescribing cascades rated as true due to insufficient clinical information.

### Comparison with previous literature

Few previous studies have examined all nine ThinkCascades dyads, focusing instead on individual prescribing cascades, or examination of larger lists of prescribing cascades identified via literature review [39, 40]. Hence drawing comparisons on the overall incidence of any ThinkCascades dyad is challenging. One study, which examined ThinkCascades occurrence within the Irish Longitudinal Study on Ageing (TILDA), identified that over nine years, 2.1% of adults aged ≥ 50 years experienced any ThinkCascades potential cascade [41]. An additional Irish study, which examined dispensed medication data for a national sample of older adults aged ≥ 65 years, found the prevalence of each individual ThinkCascades dyad range from 0.4 to 3.6% [26]. 

The CCB to diuretic prescribing cascade is perhaps the most investigated prescribing cascade examined within the literature. The one-year incidence proportion identified here (9.2%) is greater than that reported in previous Northern American and Irish studies, where between 1.4% and 4.9% of incident CCB users have been observed to commence a diuretic within one year of CCB initiation [42, 43]. However, independent assessments of the cases identified resulted in an overall positive predictive value of 23.1% which is considerably less than that reported in a previous American studied that incorporated a chart review of cases identified within hospital administrative data (54.7%) [27]. Nevertheless, it should be noted that that an assessment of true cascade likelihood could not be conducted for one case which reduced the positive predictive value.

In the current study only six out of nine ThinkCascades dyads were identified. This is similar to previous studies that examined all nine ThinkCascades dyads within Irish data and only identified evidence for five medication pairs, albeit with some subtle differences in the dyads identified [26, 41]. In addition to being identified in both Irish studies, the present study identified potential cases of the CCB to diuretic, benzodiazepine to anti-psychotic, and alpha-1-receptor blocker to vestibular sedative prescribing cascades within the Dutch context [26, 41]. Previous studies have indicated that prescribing cascade occurrence may vary internationally and so it is possible that certain ThinkCascades are less relevant when applied in different healthcare systems. A recent examination of Dutch community pharmacy data did not find evidence to support the benzodiazepine to anti-dementia medication cascade [40]. Similar findings have been reported in examinations of Irish dispensed medication and longitudinal cohort data [26, 41]. In contrast, the present study identified one case for this ThinkCascades dyad, although independent clinical assessment deemed this as prescribed for another reason and not a confirmed prescribing cascade.

Alternatively, ThinkCascades may contain indicators that are not as clinically relevant for older adults in practice. An updated indicator set of potential prescribing cascades (*n* = 65) considered of importance in older people developed by international multidisciplinary exert consensus includes only six out of nine ThinkCascades within the updated list [22]. Those ThinkCascades dyads not incorporated into this indicator set include (i) benzodiazepine to antipsychotic; (ii) SSRI/SNRI to sleep agent; and (iii) urinary anticholinergic to anti-dementia medication. However, it should be noted that the converse relationship for the latter example, acetylcholinesterase inhibitor leading to urinary anticholinergic was included in the final list [22]. 

### Implications for practice, policy and research

Reducing medication-related harm is a WHO global priority. It remains unclear to what extent prescribing cascades contribute to potentially inappropriate prescribing and problematic polypharmacy for patients. Whilst the incidence of ThinkCascades was low in the present study sample, the potential impact at a population may be greater given the widespread prescribing of these medications. Therefore, any efforts to raise awareness of prescribing cascades, such as explicit lists of prescribing cascades are warranted in order to increase awareness of this form of potentially inappropriate prescribing in older adults. Furthermore, at the patient level prescribing cascades can have deleterious consequences. It has previously been shown that older adults who experience the CCB to diuretic cascade experience higher rates of serious adverse events (emergency room visit or hospitalization) [44]. Once established prescribing cascades may continue for some time, as illustrated in a Dutch proof-of-concept study [45]. Furthermore, a considerable proportion of individuals who experience a prescribing cascade may continue the second/cascade medication after the first has been discontinued as identified in a recent US-based population study [46]. 

Prescribing decisions within the management of multimorbidity in primary care requires an integration of patient preferences and treatment goals, the best available evidence and clinical experience and expertise [47, 48]. Protected time for regular structured medication reviews targeting higher risk patients and collaborative efforts between clinical pharmacists and physicians are important health system level enablers to identify and address higher risk prescribing and medication related harm. Whilst pharmacists in The Netherlands do not currently have formal prescribing rights general practice-based pharmacists have previously been shown to work alongside their GP colleagues, using collaborative agreements about prescribing for or reissuing long-term prescriptions for certain patients that demonstrated a high degree of autonomy [49]. The integration of a clinical pharmacist into the Dutch general practice has previously been shown to result in the identification of a large number of medication-related problems, which translated into a high rate of implementation of pharmacist recommendations (83%), 78% of which completely resolved medication-related problems [50]. Furthermore, this intervention was shown to result in a significantly fewer medication-related hospitalizations, when compared with usual care [51]. Integrating explicit lists of prescribing cascades and other aspects of potentially inappropriate prescribing into GP electronic health record software systems may also offer opportunities to identify, prevent and address potentially inappropriate prescribing cascades. Nevertheless, some emerging evidence suggests that these approaches have yet to fulfill their promise [52]. 

General practice faces increasing demands in terms of delivering care to changing populations with substantially increased levels of multimorbidity and complex polypharmacy with increases in patient-facing workload [53, 54]. Furthermore, work not involving direct patient contact represents a considerable portion of the workload facing GPs [54–56]. A recent simulation study estimated that the time required for a primary care physician to deliver clinical guidelines-based recommended care to a GP practice population of 2500 patients far exceeds that of a standard working (8 h) day (26.7 h per day), and considering that most GPs have on average 8–15 min direct interaction time per patient [53, 55, 57]. Consequently, detecting and addressing potentially inappropriate prescribing cascades proffered by expanded explicit lists must be balanced with prioritizing other aspects of clinical care and outcomes that are most important to patients using a shared decision making framework [48]. 

The present study’s findings also highlight the importance of prescribing cascade validation when examining routine administrative data sources. Just over one-quarter of identified potential prescribing cascades were confirmed as prescribing cascades when corresponding patient EHRs were reviewed. Furthermore, the positive predictive value at a cascade level ranged from 0 to 100%, highlighting the importance of interrogating health record data for alternate explanations for prescribing. For example, for the CCB to diuretic cascade the diuretic was initiated for hypertension in six of the 13 potential cases identified. Whilst methods such as prescription sequence symmetry analysis (PSSA) can be efficiently employed at a large-scale level and used for hypothesis generation to detect novel prescribing cascade signals, review of more granular patient-level data is required to understand the true burden of the prescribing cascade phenomenon for patients. Consequently, several approaches may be required to expand the current evidence base on prescribing cascades (e.g., PSSA for signal detection and EHR review for cascade validation).

Furthermore, the exposure and outcome thresholds for prescribing cascade case definition may need to vary for each cascade under examination. A balance is required between the time taken for an ADR to occur and present to a prescriber for treatment, whilst minimizing the influence of time-varying confounding. As the literature on prescribing cascades continues to grow, future studies that inform of the likely window for their occurrence could support more accurate identification of prescribing cascade incidence in routine data.

### Strengths and limitations

This study has several notable strengths. It examined a list of nine potential prescribing cascades (ThinkCascades) of clinical importance in older adults which was developed by international multidisciplinary expert consensus. It utilized a comprehensive data source and harnessed both prescribing and diagnostic coding data in a Dutch database renowned for its robust data validation processes. Furthermore, it applied a relatively long observation window to examine prescribing cascades over time. Finally, the study extends the limited literature on prescribing cascade confirmation through an evaluation of the electronic health record data for each potential case via dual independent clinical assessment by a GP and a pharmacist.

However, there are some limitations. The low number of prescribing cascades events identified prevented an examination of participant characteristics associated with cascade occurrence as well any examination of subsequent secondary outcomes (e.g., healthcare utilization following cascade occurrence). It is possible that some prescriptions were not captured in the FaMe-Net data (e.g., prescriptions that would not have been electronically transferred via the national medication record system), which may have underestimated the incidence of prescribing cascades. Routine data sources often do not contain patient reported outcome measures (PROMs), which represent another important consideration when evaluating the impact of prescribing cascades. Prescribing cascades that do not lead to additional healthcare utilization can still adverse consequences for older adults through their potential to impact on functional ability and reduce quality of life. Future studies should consider evaluating PROMs and how best to integrate these patient-level outcomes with the other data sources that are necessary to identify prescribing cascades in primary care. Furthermore, prescribed medications may not be representative of true medication use at the patient level. An allowable gap in prescriptions to account for medication non-adherence or delays in obtaining prescriptions may have overestimated exposure to Drug A. However, the specification of continued use of Drug A at the point of Drug B initiation represents a closer representation of the prescribing cascade experience compared with intention-to-treat-like approaches such as used in PSSA [58, 59]. Because information on prescription medications only was available, consumption of over-the-counter medication could not be considered in this study. Finally, a contemporaneous increased international focus on addressing PIP in older adults to reduce medication-related harm may have resulted in improved prescribing practices including avoidance of prescribing cascades.

## Conclusion

Overall, this study found that prescribing cascades, as defined by ThinkCascades, were not commonly experienced by older patients attending Dutch general practice. Whilst apparently uncommon in primary care on the basis of the present study, prescribing cascades nevertheless represent an aspect of preventable medication-related harm. Given widespread challenges faced by clinicians in deprescribing medication for older adults with complex multimorbidity and polypharmacy, prevention of prescribing cascades provides an alternate angle from which to address medication-related harm. Consequently, considering ADRs as part of the differential diagnosis for older people presenting to general practice is important to optimize prescribing.

## Supplementary Information

Supplementary Material 1.

## Data Availability

Access to data used in the present study is granted upon approval of a request by the Radboudumc Technology Centres Health Centre of the Radboud university medical center Nijmegen. More information can be obtained at: https://famenet.nl/request-data/.

## Abbreviations

ADR: Adverse drug reaction
ATC: Anatomical therapeutic code
CREC: Clinical Research Ethics Committee
GP: General practitioner
ICPC: International Classification of Primary Care
PBRN: Practice-Based Research Network
PSSA: Prescription sequence symmetry analysis
