# Supplementary material for: Characterising prescribing cascades in older community-dwelling adults attending general practice in The Netherlands: a retrospective cohort study
Source: BMC Prim Care. 2026 Mar 17;27:156. doi: 10.1186/s12875-026-03273-x (PMC13107699; doi:10.1186/s12875-026-03273-x)
Supplement: Supplementary file 1 — Supplementary Material 1. [file 12875_2026_3273_MOESM1_ESM.docx]

**Additional File 1- STROBE Checklist**

|  | Item No | Recommendation | Location |
| --- | --- | --- | --- |
| **Title and abstract** | 1 | (*a*) Indicate the study’s design with a commonly used term in the title or the abstract | Abstract |
|  |  | (*b*) Provide in the abstract an informative and balanced summary of what was done and what was found | Abstract |
| Introduction | | |  |
| Background/rationale | 2 | Explain the scientific background and rationale for the investigation being reported | P4-5 |
| Objectives | 3 | State specific objectives, including any prespecified hypotheses | P5 |
| Methods | | |  |
| Study design | 4 | Present key elements of study design early in the paper | P6 |
| Setting | 5 | Describe the setting, locations, and relevant dates, including periods of recruitment, exposure, follow-up, and data collection | P6-7 |
| Participants | 6 | (*a*) *Cohort study*—Give the eligibility criteria, and the sources and methods of selection of participants. Describe methods of follow-up  *Case-control study*—Give the eligibility criteria, and the sources and methods of case ascertainment and control selection. Give the rationale for the choice of cases and controls  *Cross-sectional study*—Give the eligibility criteria, and the sources and methods of selection of participants | P7 |
|  |  | (*b*) *Cohort study*—For matched studies, give matching criteria and number of exposed and unexposed  *Case-control study*—For matched studies, give matching criteria and the number of controls per case |  |
| Variables | 7 | Clearly define all outcomes, exposures, predictors, potential confounders, and effect modifiers. Give diagnostic criteria, if applicable | P7 |
| Data sources/ measurement | 8* | For each variable of interest, give sources of data and details of methods of assessment (measurement). Describe comparability of assessment methods if there is more than one group | *P7* |
| Bias | 9 | Describe any efforts to address potential sources of bias | N/A |
| Study size | 10 | Explain how the study size was arrived at | N/A |
| Quantitative variables | 11 | Explain how quantitative variables were handled in the analyses. If applicable, describe which groupings were chosen and why | P8-9 |
| Statistical methods | 12 | (*a*) Describe all statistical methods, including those used to control for confounding | P8=9 |
|  |  | (*b*) Describe any methods used to examine subgroups and interactions | N/A |
|  |  | (*c*) Explain how missing data were addressed | N/A |
|  |  | (*d*) *Cohort study*—If applicable, explain how loss to follow-up was addressed  *Case-control study*—If applicable, explain how matching of cases and controls was addressed  *Cross-sectional study*—If applicable, describe analytical methods taking account of sampling strategy | N/A |
|  |  | (*e*) Describe any sensitivity analyses | P8-9 |

| Results | | | Location |
| --- | --- | --- | --- |
| Participants | 13* | (a) Report numbers of individuals at each stage of study—eg numbers potentially eligible, examined for eligibility, confirmed eligible, included in the study, completing follow-up, and analysed | P9 |
|  |  | (b) Give reasons for non-participation at each stage | N/A |
|  |  | (c) Consider use of a flow diagram | N/A |
| Descriptive data | 14* | (a) Give characteristics of study participants (eg demographic, clinical, social) and information on exposures and potential confounders | Table 2 |
|  |  | (b) Indicate number of participants with missing data for each variable of interest | Table 2 |
|  |  | (c) *Cohort study*—Summarise follow-up time (eg, average and total amount) | N/A |
| Outcome data | 15* | *Cohort study*—Report numbers of outcome events or summary measures over time | *Table 3* |
|  |  | *Case-control study—*Report numbers in each exposure category, or summary measures of exposure |  |
|  |  | *Cross-sectional study—*Report numbers of outcome events or summary measures |  |
| Main results | 16 | (*a*) Give unadjusted estimates and, if applicable, confounder-adjusted estimates and their precision (eg, 95% confidence interval). Make clear which confounders were adjusted for and why they were included | N/a |
|  |  | (*b*) Report category boundaries when continuous variables were categorized |  |
|  |  | (*c*) If relevant, consider translating estimates of relative risk into absolute risk for a meaningful time period |  |
| Other analyses | 17 | Report other analyses done—eg analyses of subgroups and interactions, and sensitivity analyses | Table 3 |
| Discussion | | |  |
| Key results | 18 | Summarise key results with reference to study objectives | P10 |
| Limitations | 19 | Discuss limitations of the study, taking into account sources of potential bias or imprecision. Discuss both direction and magnitude of any potential bias | P14-15 |
| Interpretation | 20 | Give a cautious overall interpretation of results considering objectives, limitations, multiplicity of analyses, results from similar studies, and other relevant evidence | P10-15 |
| Generalisability | 21 | Discuss the generalisability (external validity) of the study results |  |
| Other information | | |  |
| Funding | 22 | Give the source of funding and the role of the funders for the present study and, if applicable, for the original study on which the present article is based | P17 |

**Additional File 2-Specification of the benzodiazepine discontinuation leading to antipsychotic initiation prescribing cascade**

For the benzodiazepine to antipsychotic prescribing cascade, where the exposure was benzodiazepine discontinuation, as opposed to initiation, the first exposure period for incident benzodiazepine users was still examined. However, exclusion diagnoses were applied until the end date of the last benzodiazepine prescription during that exposure period (as opposed to the start date). A discontinuation date was set as 30 days after the end date of the last benzodiazepine prescription. A discontinuation prescribing cascade was defined as incident use of an antipsychotic within 180 days of the benzodiazepine discontinuation date. The denominator was the number of new users of benzodiazepines who had a discontinuation date prior to 1^st^ Jan 2021.

**Additional File 3- ICPC-2 codes for long-term conditions used to determine presence of multimorbidity (adapted from Ho et al. 2022)**^1^

| **Diagnosis** | **ICPC-2 code** |
| --- | --- |
| Stroke | K90, K91 |
| Coronary artery disease | K74-K76 |
| Heart failure | K77 |
| Peripheral artery disease | K92 |
| Heart valve disorders | K83 |
| Arrhythmia | K80 |
| Hypertension (treated and untreated) | K86, K87 |
| Venous thromboembolic disease | K94 |
| Diabetes | T89, T90 |
| Addison’s disease or cystic fibrosis | T99 *(Endocrine/metabolic-other*) |
| Thyroid disorders (hypothyroidism or hyperthyroidism) | T85, T86 |
| Chronic obstructive pulmonary disease | R85 |
| Asthma | R96 |
| Parkinson’s disease | N87 |
| Epilepsy | N88 |
| Multiple sclerosis | N86 |
| Paralysis | N18 |
| Transient ischaemic attack | K89 |
| Peripheral neuropathy | N84 |
| Solid organ cancers | A79, D74-D77, K72, N74, R84-85, T71, U75-77, X75-77, Y77-78 |
| Haematological cancers | B72, B73 |
| Dementia | P70 |
| Schizophrenia | P72 |
| Depression | P76 |
| Anxiety | P74 |
| Affective psychosis | P73 |
| Substance abuse | P15, P16, P19 |
| Post-traumatic stress disorder | P82 |
| Rheumatoid arthritis | L88 |
| Osteoarthritis | L89-L91 |
| Osteoporosis | L95 |
| Chronic liver disease | D97 |
| Inflammatory bowel disease | D92, D94 |
| Chronic kidney disease | U88 *(Glomerulonephritis/nephrosis)* |
| End stage kidney disease | U99 *(Urinary disease-other)* |
| HIV/AIDS | B90 |

**References**

1. Ho ISS, Azcoaga-Lorenzo A, Akbari A, et al. Measuring multimorbidity in research: Delphi consensus study. *BMJ Medicine*. 2022;1(1):247. doi:10.1136/BMJMED-2022-000247
